# Texture Analysis of Polycrystalline Vaterite Spherulites from Lake Sturgeon Otoliths

**DOI:** 10.1038/s41598-019-43434-w

**Published:** 2019-05-09

**Authors:** Bryan C. Chakoumakos, Brenda M. Pracheil, R. Seth Wood, Alison Loeppky, Gary Anderson, Ryan Koenigs, Ronald Bruch

**Affiliations:** 1 Neutron Scattering Division, Oak Ridge National Laboratory, Oak Ridge, TN 37831 USA; 2 Environmental Sciences Division, Oak Ridge National Laboratory, Oak Ridge, TN 37831 USA; 3 Department of Earth & Planetary Sciences, University of Tennessee, Knoxville, TN 37996 USA; 4 Department of Biological Sciences, University of Manitoba, Winnipeg, MB R3T 2N2 Canada; 5 Wisconsin Department of Natural Resources, Oshkosh, WI 54901 USA

**Keywords:** Applied physics, Biomineralization

## Abstract

Fish otoliths, or ear bones, are comprised of the CaCO_3_ polymorphs (aragonite, calcite and vaterite), which can occur either alone or in combination. The polymorph phase abundance in an otolith depends on, as yet, unexplained genetic and environmental factors. Most fish otoliths are comprised of the densest CaCO_3_ polymorph, aragonite. Sturgeon otoliths, on the other hand, contain significant amounts of the rare and the structurally enigmatic polymorph, vaterite. Sturgeon otoliths are frequently comprised of agglomerations of small microcrystalline vaterite spherulites (<300 *μ*m in diameter), that range in shape from nearly perfect spheres to oblate spheroids. These spherulites are similar to the synthetic vaterite microspheres employed in laser trapping applications. Vaterite spherulites from both hatchery-reared (juvenile) and wild (adult) Lake Sturgeon exhibit extreme crystallographic texture as evidenced by X-ray diffraction patterns and their reconstructed pole-figures determined here. The vaterite crystallites making up the spherulites have excellent registry in both the axial and equatorial directions. Whether synthesized or natural, the texture manifested in these spherulites suggests that vaterite nucleates and grows similarly *in vivo* otolith formation as well as from laboratory synthesis. The uniaxial optical character of the vaterite spherulites, confirmed by these diffraction experiments and combined with their large birefringence, makes them well suited for laser trapping applications.

## Introduction

In the course of our studies of fish otoliths, we have noticed microstructural habits of vaterite that appear to be identical to synthetically grown vaterite that is used in optical trapping devices to manipulate and measure properties of fluids in small volumes. Fish otoliths, or ear bones, are comprised of the CaCO_3_ polymorphs (aragonite, calcite and vaterite), which can occur either alone or in combination. Three pairs of otoliths occur in finfish (class Osteichthyes), the sagittae, lapilli, and asterisci. The sagittae, which are typically the largest pair and found just behind and approximately vertically level to the eyes, are most often comprised of the densest CaCO_3_ polymorph, aragonite. The typically smaller lapilli and asterisci are located within the semicircular canals and are often comprised of vaterite. However, individual otoliths can also be made up of more than one CaCO_3_ polymorph, and the polymorph phase abundance can be variable among individual fish, and even between the otolith pairs in individual fish^1^. The polymorph phase abundance in an otolith depends on, as yet, unexplained genetic and environmental factors. The sagittal otoliths of Lake Sturgeon (*Ascipenser fulvescens*) contain significant amounts of the rarer and structurally enigmatic polymorph, vaterite^2^. Lake Sturgeon otoliths are frequently comprised of agglomerations of small microcrystalline vaterite spherulites (<100 μm in diameter), that range in shape from nearly perfect spheres to oblate spheroids. The lapilli otoliths of larval and juvenile Lake Sturgeon can consist of a single spherulite of vaterite^3^. These spherulites are similar to synthetic vaterite microspheres frequently employed in microrheological systems used to measure properties of complex fluids in small fluid volumes. In these instruments, optical tweezers^4^ are employed to trap and rotate the birefringent vaterite microspheres, and their diffusional rotation is relatable to the fluid viscosity. Whether synthesized or natural, the texture manifested in these microspheres gives clues to how vaterite nucleates and grows.

## Results and Discussion

**Figure 1:**
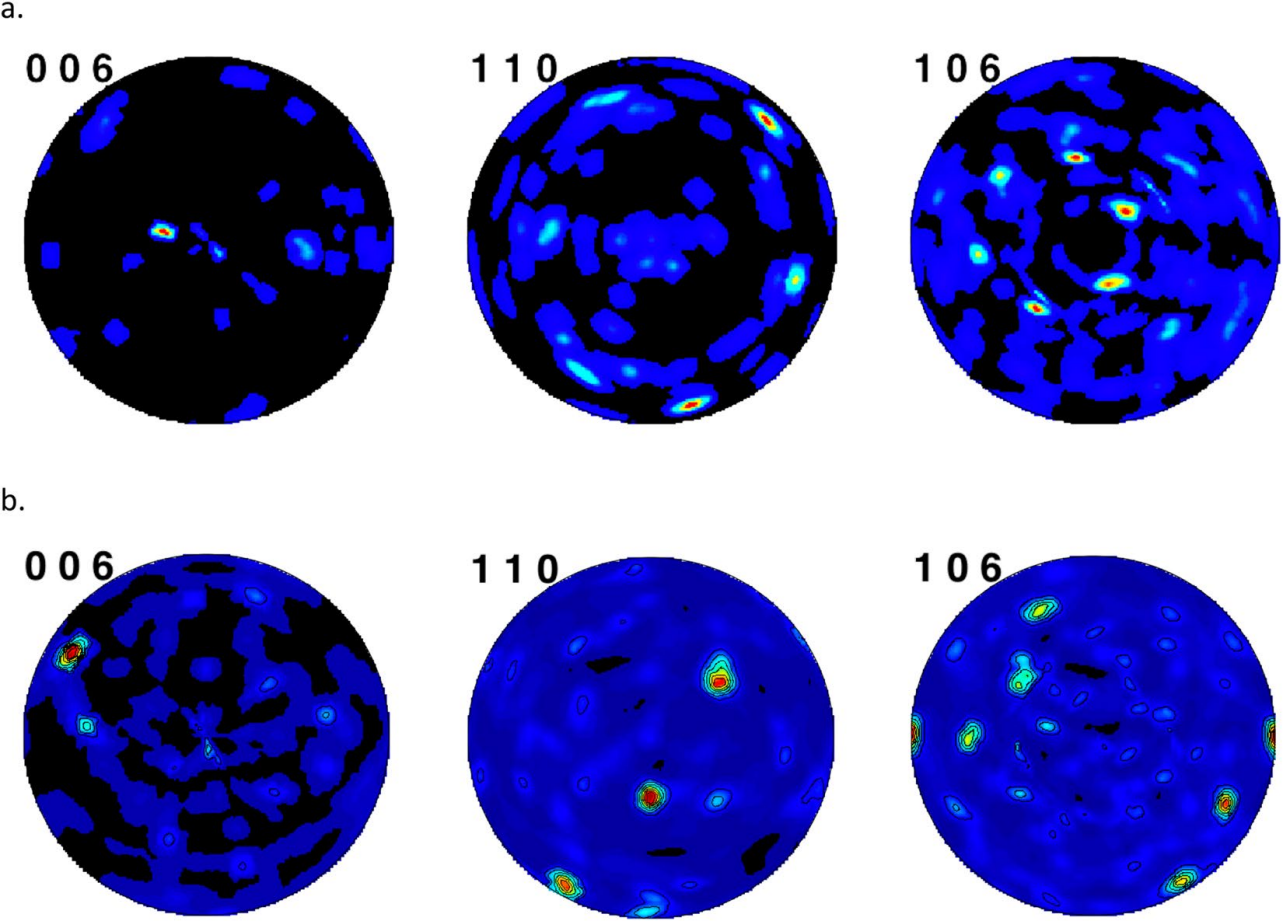
Reconstructed pole figures for vaterite spherulites from (**a**) hatchery-reared juvenile Lake Sturgeon otolith, University of Manitoba (**b**). Adult Lake Sturgeon otolith, Lake Winnebago, Wisconsin. Colors represent the frequency of the indicated crystallographic directions, (006), (110), and (106), and their equivalents, with the hot colors showing the highest frequency.

Both spherulites from both hatchery-reared (juvenile) and wild Lake Sturgeon (adult) exhibit extreme crystallographic texture. The overall agreements of the Rietveld fits were *R*_p_ = 3.46% and 3.06%, respectively. The reconstructed pole figures show nearly single crystal like appearance with the crystallites having excellent registry in both the axial and equatorial directions (Fig. 1). Keep in mind that these spherulites were arbitrarily mounted for the diffraction data collections. Their uniaxial optical character would be evident using polarized light microscopy, but otherwise their preferred orientation axis cannot be easily discerned. Diffraction patterns of several vaterite spherulites larger than 100 *μ*m from Lake Winnebago Lake Sturgeon showed the crystallographic preferred orientation to degrade for these larger spherulite sizes, which is generally consistent with the observation by Parkin *et al*.^5^ that the optical retardation of synthetic vaterite spherulites increases with diameter but reaches a plateau beyond a diameter of ~10 microns.

## Conclusions

We conjecture that vaterite nucleation and growth habit from synthetic recipes^5,6^ and *in vivo* Lake Sturgeon otolith formation are similar, resulting in the formation of spherulites in which the component crystallites show strong subparallel preferred orientation. This contrasts with the more common spherulitic growths associated with radiating or concentric textures.

**Figure 2:**
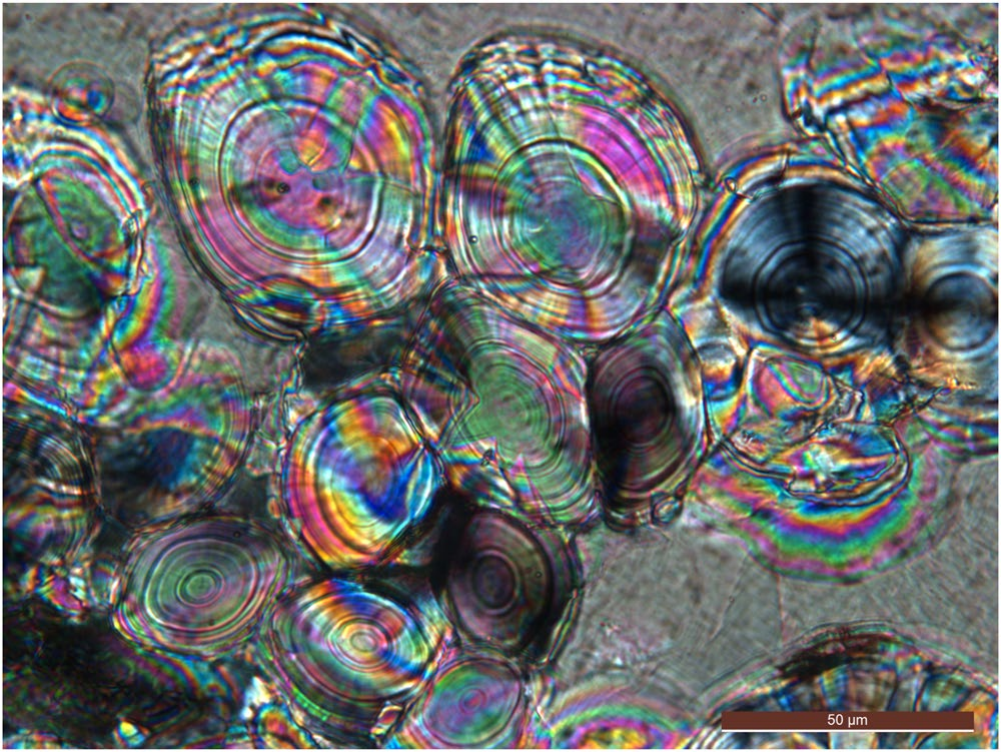
Optical micrograph (crossed polarizers) of vaterite spherulites in a matrix of a large calcite crystal from an adult Lake Sturgeon otolith, Lake Winnebago, Wisconsin (standard polished thin section 30 μm thick). The onion-like rings of the spherulites are interpreted to be the daily growth rings. One spherulite in the right center is oriented such that the isogyres of a uniaxial interference figure are seen.

X-ray diffraction of juvenile and adult vaterite-rich Lake Sturgeon otoliths, constituting single spherulites, exhibit pronounced crystallographic preferred orientation, despite that the vaterite spherulites from Lake Sturgeon otoliths exhibit daily growth layers (Fig. 2). The uniaxial optical character of vaterite spherulites is consistent with a sheaf-like texture, and this single crystal like form enables them to efficiently couple with laser light in laser trapping applications^7,8^. Fish otoliths themselves are an example where optical trapping has been used to move 55 μm size aragonite otoliths of larval zebra fish *in vivo* to stimulate and map the functional neural connections to the fish’s body^9^.

## Methods

### Samples

Sagittal otoliths from adult Lake Sturgeon used in this study were voluntarily contributed by state-licensed anglers in a sustainably-managed Lake Sturgeon sport fishery on Lake Winnebago, Wisconsin. Worldwide, many sturgeon species are either threatened or endangered, however, the Lake Winnebago sturgeon fishery has been sustainably managed for more than a century^10^. No live animals from Lake Winnebago were handled by the authors for the purposes of this study. A survey of sagittal otoliths from a number of adult Lake Sturgeon of the Lake Winnebago fishery show them to be primarily vaterite (~80 wt%) with some calcite (~20 wt%)^5^. The vaterite in these otoliths often occurs as spherulites ranging from <1 to 300 μm (Fig. 2). For this study, a spherulite ~30 *μ*m in diameter was used. In thin section (30 μm thick), this vaterite is uniaxial (+) and shows 5^th^ order interference colors corresponding to a birefringence of ~0.08, which compares reasonably well with the range of 0.094–0.100 reported in mineralogical databases^11^.

A second sample used in this study was from a juvenile fish, hatchery-reared by one of the authors (AL) using eggs and milt from wild fish of the Winnipeg River system in northern Manitoba, Canada. This lapilli otolith consisted of a single spherulite (~100 μm) of vaterite from an individual 78 days old^4^. All procedures conducted on these fish were approved by the Animal Care Committee at the University of Manitoba permit# F15-007 in accordance with guidelines established by the Canadian Council for Animal Care.

### X-ray diffraction

**Figure 3:**
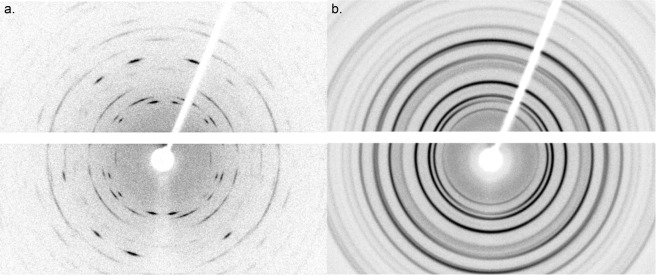
X-ray diffraction patterns (Mo Kα radiation): (**a**) of a lapilli otolith consisting of a single vaterite spherulite (~100 μm in diameter) from a juvenile hatchery-reared Lake Sturgeon, University of Manitoba. (**b**) Ideally random powdered vaterite from a Lake Sturgeon otolith, Lake Winnebago, Wisconsin. The images were recorded for 300 s while rotating the samples 1°/s around the vertical *ϕ* axis. The detector consists of 2 segments with a horizontal dead space between them. The shadow of the beam stop extends upward to the right. A faint shadow of the sample mounting pin extends down from center.

**Figure 4:**
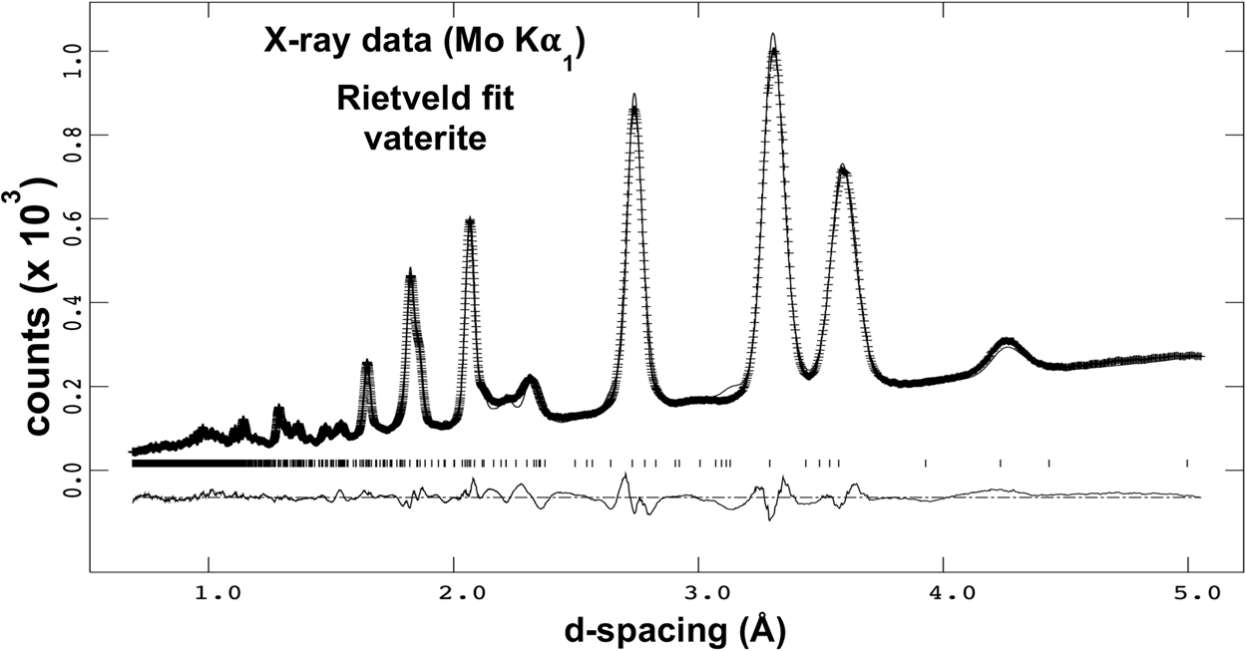
Rietveld refinement fit of ideally random powdered vaterite spherulites from an adult Lake Sturgeon otolith, Lake Winnebago, Wisconsin. Crosses are the experimental data, the solid line is the model fit, the vertical bars mark the reflection peak positions, and the lower curve is the difference between the model and the observed intensities. The *P*6_5_22 space group model of Wang and Becker^19^ was used, and although not perfect, it is sufficient for analyzing the texture model.

Individual spherulites were mounted in the manner of single crystals, using a 300 *μ*m diameter Molecular Dimensions LithoLoop with a drop of Paratone oil. Data were collected using a Rigaku XtaLAB PRO diffractometer equipped with graphite monochromated Mo Kα radiation, a Dectris Pilatus 200 K detector, and the Rigaku Oxford Diffraction CrysAlisPro software. The flat-plate detector center, distance, and orientation, as well as the peak shape parameters were calibrated using the NIST LaB_6_ Standard Reference Material 660C powder on the same style mounting loop. Crystallographic texture is manifested in the diffraction pattern by incomplete Debye-Scherrer rings (Fig. 3). Data for the texture analysis of the vaterite spherulites were collected at 7 *ω* angles −60°, −40°, −20°, 0°, +20°, +40° and +60° for 2*θ* = 0°. Each image was recorded for 300 s, for a total data collection time of 35 mins per sample. Each of these detector images was processed into radial scans every 5° of η (angular coordinate around the diffraction rings). Those scans that intercepted the beam-stop shadow or the dead-space of the detector segments were not used. Texture analysis was determined by Rietveld refinement^12^ with the software package Materials Analysis Using Diffraction (MAUD 2.78)^13^ using the WIMV method (see review by Matthies *et al*.^14^) and following the analysis procedure of Lutterotti *et al*.^15^. The orientation distribution function resolution used was 5°. Although the exact crystal structure of vaterite has been the subject of discussion for over 50 years, see reviews by Christy^16^, Makovicky^17^, and Wang *et al*.^18^, we have adopted the *P*6_5_22 model proposed by Wang and Becker^19^ which gives a sufficiently good fit to both neutron and X-ray diffraction data^20,21^ (Fig. 4). The structural parameters were held fixed during the refinement, but the background, scale factors, and sample shifts were refined. The data for at least 3 values of *ω* angles were used in the final analysis to ensure sufficient pole figure coverage. The datasets generated during and analysed during the current study are available from the corresponding author on reasonable request.
